# Conditional deletion of neurexin-2 impaired behavioral flexibility to alterations in action–outcome contingency

**DOI:** 10.1038/s41598-024-60760-w

**Published:** 2024-05-03

**Authors:** Sheraz Khoja, Lulu Y. Chen

**Affiliations:** 1 Department of Anatomy and Neurobiology, School of Medicine, University of California, Irvine, CA 92697 USA; 2 Center for Neurobiology of Learning and Memory, Herklotz Research Facility, University of California, Irvine, CA 92697 USA

**Keywords:** Molecular neuroscience, Habituation

## Abstract

Neurexins (Nrxns) are critical for synapse organization and their mutations have been documented in autism spectrum disorder, schizophrenia, and epilepsy. We recently reported that conditional deletion of *Nrxn2*, under the control of *Emx1Cre* promoter, predominately expressed in the neocortex and hippocampus (*Emx1-Nrxn2* cKO mice) induced stereotyped patterns of behavior in mice, suggesting behavioral inflexibility. In this study, we investigated the effects of *Nrxn2* deletion through two different conditional approaches targeting presynaptic cortical neurons projecting to dorsomedial striatum on the flexibility between goal-directed and habitual actions in response to devaluation of action–outcome (A–O) contingencies in an instrumental learning paradigm or upon reversal of A–O contingencies in a water T-maze paradigm. *Nrxn2* deletion through both the conditional approaches induced an inability of mice to discriminate between goal-directed and habitual action strategies in their response to devaluation of A–O contingency. *Emx1-Nrxn2* cKO mice exhibited reversal learning deficits, indicating their inability to adopt new action strategies. Overall, our studies showed that *Nrxn2* deletion through two distinct conditional deletion approaches impaired flexibility in response to alterations in A–O contingencies. These investigations can lay the foundation for identification of novel genetic factors underlying behavioral inflexibility.

## Introduction

Neurexins (Nrxns) are presynaptic cell adhesion molecules that have well established roles in regulating synapse assembly^1–3^, assembly of presynaptic release machinery^1,2,4–8^, and postsynaptic neurotransmission^2,5,6,9^. The mammalian genome contains three genes (*Nrxn1*, *Nrxn2*, *Nrxn3* in mice; and *NRXN1*, *NRXN2*, *NRXN3* in humans). *NRXN* mutations have been widely documented in autism spectrum disorder (ASD)^10–14^, epilepsy^15–17^, schizophrenia^10,18,19^, and Tourette syndrome^20^. Hence, generation of genetic mouse models play an invaluable role in the exploration of biological mechanisms by which *NRXN* mutations can contribute to the pathophysiology of these neuropsychiatric disorders.

Constitutive and conditional deletion for *Nrxn2* induced stereotyped patterns of behavior^21,22^ and reversal learning deficits^23^ in mice. However, previous findings did not address the adaptability of *Nrxn2* cKO mice to shift between distinct action selection strategies in an environment where changes in circumstances can alter the value of those actions. The observed stereotypy and inflexible nature in these genetically modified mice forms the basis for our hypothesis that *Nrxn2* deletion disrupts the learning processes integral to guiding action control in a dynamic environment. In this study, we aim to address the following questions: (1) Does *Nrxn2* deletion alter the rate of habit formation? or (2) Does *Nrxn2* deletion disrupt the ability to assess the value of consequences of an action and to use that information to guide action control when those actions become devalued? (3) Does *Nrxn2* deletion from corticostriatal circuits implicated in goal-directedness disrupt the shift between goal-directed and habitual actions?

**Figure 1:**
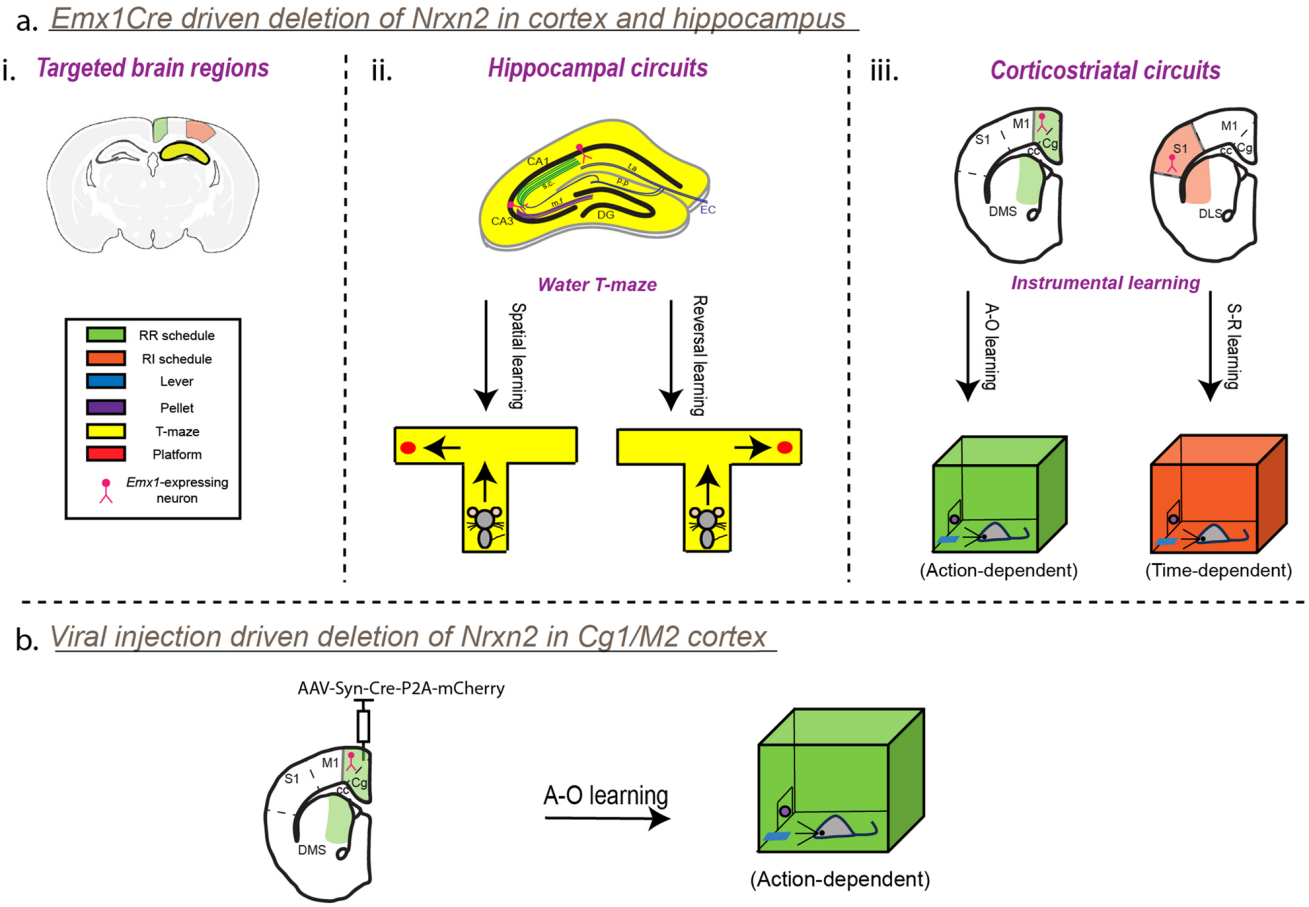
Circuit-specific regulation of two discrete behavioral flexibility paradigms. (**a**) i) A schematic depicting *Emx1Cre* driven deletion of *Nrxn2* in the neocortex and hippocampus. *Nrxn2*^*f/f*^ mice were crossed with *Emx1Cre* mice to target pyramidal neurons (colored in pink) in the neocortex (colored in green for associative cortex and in orange for sensorimotor cortices) and in the hippocampus (colored in yellow). (ii) Water-T-maze (colored in yellow) was used to assess the ability of *Emx1-Nrxn2* cKO mice to find the platform (colored in red) through integration of spatial cues with reversal of action-outcome (A–O) contingencies. (iii) The Cg1/M2-DMS circuitry (colored in green) is involved in A–O learning and S1/M1-DLS circuitry (colored in orange) is involved in stimulus-response (S–R) learning. (**b**) A schematic showing adeno-associated virus (AAV) injection driven deletion of *Nrxn2* in the associative neocortex (Cg1/M2) of *Nrxn2*^*f/f*^ mice.

Actions can be controlled by two distinct learning systems: the action–outcome (A–O) system and the stimulus–response (S–R) system. The A–O learning system is driven by the anticipated value of the reward, making it goal-directed in nature. On the other hand, S–R system is not influenced by the anticipated value of reward but by associated stimuli, rendering it habitual in nature^24–26^. Goal-directed actions compete with habits for control, and this balance forms the underlying basis for our behavioral flexibility in a dynamic environment^27^. The A–O and S–R learning behaviors are governed by two distinct corticostriatal circuits. The dorsal striatum is an anatomically heterogenous structure that can be divided into the dorsomedial striatum (DMS) and dorsolateral striatum (DLS), both of which receive inputs from different cortical regions and serve discrete behavioral functions^28,29^. The DMS receives projections from the associative cortices, including the anterior cingulate cortex (Cg1) [part of the medial prefrontal cortex (mPFC)] and the premotor cortex (M2)^30,31^. Multiple strategies, including excitotoxic lesions or reversible inactivation through muscimol infusions, have shown the DMS to be implicated in goal-directed actions^32,33^ [Fig. 1a(iii)]. The DLS receives projections from the sensorimotor cortex (M1/S1)^30^ and is implicated in S–R behaviors^34–36^ [Fig. 1a(iii)]. Disruption of synaptic activity in either of these circuitries can lead to the over-reliance on habitual actions at the expense of goal-directed actions, resulting in behavioral inflexibility.

A suitable behavioral paradigm for investigating the shift between goal-directed and habitual actions is the instrumental lever pressing paradigm. Rodents can be subjected to different schedules of reward delivery that can favor distinct action control strategies, either goal-directed or habitual. The two major types of reinforcement schedules are random interval (RI) and random ratio (RR). In the RI schedule, rewards will be delivered after a specified interval of time after the first lever press with a predetermined probability of reinforcement, promoting habitual behavior. Conversely, in the RR schedule, rewards will be delivered after a specified number of lever press with a predetermined probability of reinforcement, favoring goal-directedness^27,37,38^. To determine if the instrumental behavior of rodents is governed by goal-directed or habitual actions, an outcome devaluation test is introduced after sufficient training in the RI and RR schedules^37,39^. Habitual behaviors are insensitive whereas goal-directed behaviors are sensitive to outcome devaluation.

In this study, we employed two different mouse models in which *Nrxn2* gene is conditionally deleted from presynaptic cortical neurons projecting into the DMS. In the first mouse model, *Nrxn2* is deleted under the control of *Emx1Cre* promoter (denoted as “*Emx1-Nrxn2* cKO” mice) that is predominately expressed in excitatory neurons in neocortex and hippocampus [Fig. 1a(i)]. In the second mouse model, *Nrxn2* is deleted in the Cg1/M2 cortex (denoted as “Cg1/M2 *Nrxn2* cKO” mice) through viral-mediated injection of Cre recombinase in *Nrxn2*^*f/f*^ mice (Fig. 1b). Both of these genetically modified mice were subjected to a dual contextual instrumental lever pressing paradigm in which mice were trained to press a lever to receive a food reward under training schedules that promotes goal-directed or habitual strategies followed by an outcome devaluation test.

The *Emx1-Nrxn2* cKO mice failed to discriminate between RI and RR schedules of reinforcement as their instrumental behavior was sensitive to outcome devaluation in both contexts. Cg1/M2 *Nrxn2* cKO mice failed to distribute their lever presses in response to outcome devaluation in the RR context. Due to failure of *Emx1-Nrxn2* cKO mice to differentiate between contexts that promote distinct action selection strategies, we also conducted a water T-maze test in *Emx1-Nrxn2* cKO mice to assess reversal learning [Fig. 1a(ii)]. Similar to previous findings, *Emx1-Nrxn2* cKO mice exhibited reversal learning deficits indicating their inability to adjust to new spatial contexts and learn new behavioral actions. Overall, our studies provide novel insights into mechanisms by which *Nrxn2* deletion can lead to behavioral inflexibility.

## Methods

### Animals

Mice were maintained on a C57BL/6J background and obtained from Jackson laboratories. *Emx1-Nrxn2* cKO mice were generated and genotyped as described previously^21^. Mice were maintained on a 12/12 h light/dark cycle with ad libitum access to water and a high-fat chow. Male WT and *Nrxn2* cKO littermates were weaned at P21 and were used for behavioral tests at 2–4 months of age. For the behavioral experiments, separate cohorts of mice were used for instrumental learning paradigm and water T-maze. All procedures were conducted in accordance with the National Institute of Health Guide for the Care and Use of Laboratory Animals and protocols approved by the Institutional Animal Care and Use Committee of the University of California Irvine, including efforts to minimize suffering and the number of animals used. All animal studies were undertaken in accordance with ARRIVE guidelines.

### Stereotaxic injections

*Nrxn2*^*f/f*^ mice were injected with adeno-associated (AAV) viruses bilaterally into the Cg1/M2 region at P35-55 using a stereotaxic apparatus (Kopf Instruments, Tujunga, CA) under ketamine (80 mg/kg)–xylazine (7.3 mg/kg) induced anesthesia. 0.5 µl of concentrated virus (10^7^ transduction units per ml) was injected at a rate of 15 µl/min using a syringe pump (Harvard Apparatus, Holliston, MA) and injection needle was withdrawn 10 min post injection. Mice were injected with AAV-Syn-Cre-P2A-mCherry or AVV-Syn-ΔCre-P2A-mCherry at 2 sites per hemisphere (total = four injection sites; coordinates = i) Anterior/posterior: 1.8 mm, medial/lateral: ± 0.5 mm, dorsal/ventral: 1.7–0.7 mm; ii) Anterior/posterior: 1.18 mm, medial/lateral: ± 0.5 mm, dorsal/ventral: 1.7–0.7 mm, distance measured from Bregma). WT and Cg1/M2 *Nrxn2* cKO mice underwent context-dependent instrumental learning at 4 weeks post injection. At the end of the behavioral experiment, mice were anesthetized with isoflurane and perfused transcardially with 0.9% saline followed by 4% paraformaldehyde solution. The brains were stored in 20% sucrose solution for 2 days before being sliced into 100 µm-coronal sections through the striatum. Brain sections were mounted on microscope slides and imaged using a Keyence microscope (BZ-X800) to verify injection site and mCherry expression.

### Instrumental learning paradigm

#### Behavioral apparatus

The balance between goal-directed and habitual behaviors was investigated by using two identical operant chambers (Med-Associates, St. Albans, VT), enclosed in sound attenuating boxes that were differentiated by contextual cues (15 mm wide multi-colored washi tape that was vertically and horizontally aligned on chamber walls to give a checkerboard-like pattern or clear plexiglass chamber walls). Each chamber was equipped with a pellet dispenser that delivered 20 mg food pellets (Bio-Serv, Flemington, NJ) into a recessed food magazine, two retractable levers on either side of the food magazine, a house light, a stainless-steel grid floor and an 8 input/16 output connection panel that serves as an interface between the chambers, and a computer that runs the MED-PC V software (Med-Associates, St. Albans, VT) to operate the experimental paradigms and record lever pressing behavior. Prior to training, mice were handled, and food restricted to 85–90% of their baseline body weight. Their body weights were maintained within this range during the entirety of the instrumental learning paradigm.

#### Context-dependent training paradigm

Behavioral training and testing were conducted as previously described^40,41^. 8 mice/genotype and 5 mice/genotype were used to determine the effects of *Emx1Cre* driven and Cg1/M2-specific deletion of *Nrxn2* respectively on instrumental responding and outcome devaluation. Briefly, following 3 days of handling and food restriction, mice underwent an instrumental learning procedure in two separate contexts that lasted for 9 sessions for *Emx1-Nrxn2* cKO mice and 10 sessions for Cg1/M2 *Nrxn2* cKO mice. Each training session commenced with switching on of the house light and extension of a single lever and ended following completion of the instrumental learning task or after 60 min with the lever withdrawn and switching off of the house light. The lever position, order of context exposure, and schedule order were constant for each mouse throughout the entire training period and outcome devaluation test and counterbalanced between mice. On day 4, mice underwent a random time (RT) schedule in each context in which a food pellet was delivered on an average of 60 s in the absence of levers. This session continued for 15 min or when 15 reinforcers were delivered. Following the RT schedule, on the same day, mice were trained to press the lever under a continuous ratio of reinforcement (CRF) in each context to receive a reward. *Emx1-Nrxn2* cKO mice (and their respective control group) underwent CRF1 (1 lever press = 1 reward) on days 4–5, with the number of earned reinforcers increasing on each day (i.e., 5 reinforcers on day 4, 15 and then 30 reinforcers on day 5). Cg1/M2 *Nrxn2* cKO mice (and their respective control group) underwent CRF1 on day 4, CRF5 (5 lever presses = 1 reward) on day 5, and CRF15 (15 lever presses = 1 reward) on day 6. The maximum number of rewards was 15 for all CRF sessions for the Cg1/M2 *Nrxn2* cKO mice and their control group. All the CRF sessions continued for a total period of 60 min or when the maximum number of reinforcers was earned. Following the acquisition of lever pressing behavior, mice underwent random interval (RI) and random ratio (RR) schedules of reinforcement that were differentiated by contexts from days 6 to 11. On days 6 and 7, mice were trained to press the lever under RI30, wherein the passing of 30 s had a 15% probability of pellet dispensation and RR10, in which every lever press had a 10% chance of pellet dispensation. This was followed by RI60 and RR20 on days 8–11. All the RI and RR sessions ended once 60 min had elapsed or 15 reinforcers were dispensed. Following the RI & RR sessions, mice were provided with 1 h access to a 20% sucrose solution in their home cage as a satiety control for the outcome devaluation test.

### Outcome devaluation test

This procedure was undertaken on days 12 and 13 of the post training phase. On the valued day, mice had 1 h ad libitum access to a 20% sucrose solution in their home cage, followed by brief, non-reinforced test sessions for 5 min in both RI and RR contexts. On the devalued day, mice had 1 h ad libitum access to 20 mg food pellets that were previously earned in the operant chambers, followed by brief, non-reinforced test sessions for 5 min in both the RI and RR contexts. The order of devaluation was counterbalanced between mice.

### Water T-maze

Spatial learning and reversal learning was assessed by the water T-maze paradigm^23,41^. 6 WT and 9 *Emx1-Nrxn2* cKO mice were used for this experiment. The T-maze was filled with 20 °C (± 1 °C) water to a depth of 13 cm which is 1 cm above the surface of the platform. On the first day (pre-training phase), mice were allowed to swim freely in the T-maze without the platform for 60 s and the first arm that was chosen by the mouse was recorded. On all the training days, the platform was placed in the arm opposite to the one chosen during pre-training to avoid potential individual bias. The training session began 24 h after the pre-training phase in which the mice were given 10 trials per day with 7–10 min of rest in between each trial. Mice were placed in the start arm and given 60 s to find the platform. Once the platform was found, the mice were forced to stay on the platform for 5 s. If they were not able to find the platform within 60 s, they were gently guided to the platform and forced to stay on it for 10 s. Mice were charged with errors if (1) they left the start arm and entered the arm that does not contain the platform or the start arm or (2) they entered the arm with the platform but left that arm without staying on the platform. A trial was considered successful when the mouse left the start arm and entered the arm with the platform and stayed on it. The training session ended and the reversal learning phase began when the mice reached the criteria of 8 successful trials or greater for two consecutive days. For the reversal learning phase, the platform was placed in the arm opposite to the one chosen during the training session, and the same procedure for charging errors and determining a successful trial was followed as described above. The number of incorrect arm entries, success rate (percent of trials without an error) and days to learn spatial task were calculated for the spatial learning phase. Number of incorrect arm entries, success rate, time to reach platform and total number of trials to consistently learn the reversal task were calculated for the reversal learning phase.

### Statistical analyses

Repeated measures (RM) ANOVA was used to analyze instrumental learning and outcome devaluation data within each genotype by using day and context as repeated and independent variables respectively. RM ANOVA was also used to analyze instrumental learning between groups by using day and genotype as repeated and independent variables respectively. Reversal learning data was analyzed by RM ANOVA by using day and genotype as repeated and independent variables respectively. All RM ANOVA analyses with significant interaction between repeated and independent variables were followed by Sidak’s post hoc test for multiple comparisons. Shift in devaluation index between RI and RR schedules within each genotype for the outcome devaluation studies and number of days to learn spatial task or trials to learn the reversal task for the water T-maze test was analyzed by Student’s *t*-test. To investigate the within-subject distribution of lever presses between valued and devalued states, the number of lever presses for valued and devalued states was normalized to the total number of lever presses for both states (valued + devalued) in each context. This was followed by conducting a one sample t-test to determine whether the normalized value of lever presses in each state significantly differed from a hypothetical mean of 0.5, which normally reflects equal distribution of lever presses between valued and devalued states. A devaluation index was calculated by using the formula: [(valued presses − devalued presses)/total number of lever presses] in each context. All data were expressed as mean ± standard error of the mean (SEM). Number of mice were indicated inside bars or by their respective plots. Significance was set at *p* < 0.05. All data were analyzed by GraphPad Prism software (San Diego, CA).

## Results

### *Emx1Cre* driven deletion of *Nrxn2* did not affect instrumental responding behavior in mice

**Figure 2:**
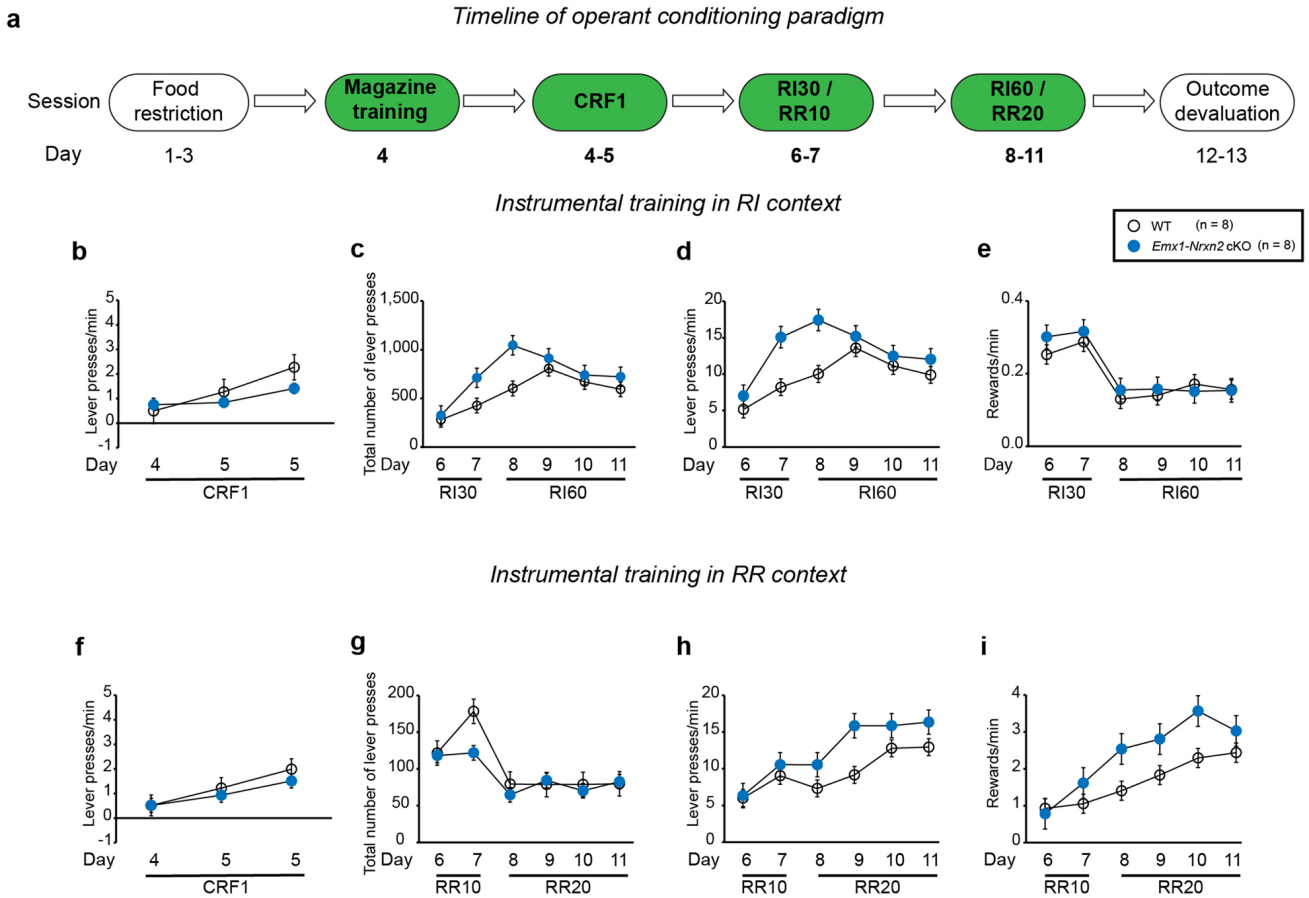
*Emx1-Nrxn2* cKO mice exhibited normal instrumental responding behavior. (**a**) Experimental timeline of instrumental training in mice. Mice underwent magazine training on day 4. This was followed by continuous ratio of reinforcement (CRF) sessions on days 4 and 5. Mice underwent CRF1 on days 4–5 with number of maximum reinforcers increasing each day (i.e., 5 reinforcers on day 4, 15 reinforcers on day 5, and 30 reinforcers on day 5). Following CRF1 sessions, mice underwent random interval (RI) and random ratio (RR) schedules of reinforcement on days 6–11 in a context-specific manner. On days 6–7, mice pressed the lever under RI30, wherein passing of 30 s had a 15% probability of pellet dispensation, and RR10 wherein every lever press had a 10% probability of pellet dispensation. This was followed by RI60 and RR20 sessions on days 8–11. Following the RI and RR sessions, mice were provided with ad libitum access to a 20% sucrose solution in their home cage. (**b**–**e**) *Emx1-Nrxn2* cKO mice exhibited normal instrumental responding behavior in the RI context. *Emx1-Nrxn2* cKO did not exhibit any significant changes in response rate (lever presses/min) [F (1,14) = 1.258, *p* = 0.2808] during the CRF sessions (**b**), total number of lever presses [F (1,14) = 0.6249, *p* = 0.4424] (**c**), response rate (lever presses/min) [F (1,14) = 0.7990, *p* = 0.3865] (**d**), and reward rate (rewards/min) [F (1,14) = 0.9837, *p* = 0.3381] (**e**) during the RI sessions. (**f**–**i**) *Emx1-Nrxn2* cKO mice exhibited normal instrumental responding behavior in the RR context. *Emx1-Nrxn2 *cKO mice did not exhibit any significant changes in response rate (lever presses/min) during the CRF sessions [F (1,14) = 0.6432, *p* = 0.4320] (**f**), total number of lever presses [F (1,14) = 5.128, *p* < 0.05] (**g**), response rate (lever presses/min) [F (1,14) = 1.133, *p* = 0.3051] (**h**), and reward rate (rewards/min) [F (1,14) = 2.060, *p* = 0.1732] (**i**) during the RR sessions. Each circle in the time-course plots (**b**–**i**) represents means ± SEM for 8 mice per genotype. Statistical assessments were performed by RM two-way ANOVA (**b**–**i**). The green color in the experimental timeline (**a**) corresponds to the operant conditioning data being represented in (**b**–**i**).

WT and *Emx1-Nrxn2* cKO mice underwent RI (to promote habitual behavior) and RR (to promote goal-directed behavior) in two separate contexts (Fig. 2a). Upon doing within-group statistical comparisons in WT mice, there was no significant effect of context [RM two-way ANOVA: F (1,14) = 0.09266, *p* = 0.7653] on response rate during the CRF1 sessions. There was a significant effect of context on total number of lever presses [RM two-way ANOVA: F (1,14) = 11.64, *p* < 0.01], on reward rate [RM two-way ANOVA: F (1,14) = 25.33, *p* < 0.001] but not on response rate [RM two-way ANOVA: F (1,14) = 0.002297, *p* = 0.9625] during the scheduled reinforcement phase in WT mice. Upon doing within-group statistical analyses in *Emx1-Nrxn2* cKO mice, there was no significant effect of context on response rate [RM two-way ANOVA: F (1,14) = 0.001892, *p* = 0.9659] during the CRF1 sessions. There was a significant effect of context on total number of lever presses [RM two-way ANOVA: F (1,14) = 12.68, *p* < 0.01], on reward rate [RM two-way ANOVA: F (1,14) = 27.21, *p* < 0.001] but not on response rate [RM two-way ANOVA: F (1,14) = 0.02380, *p* = 0.8796] during the scheduled reinforcement phase in *Emx1-Nrxn2* cKO mice.

Upon doing between-group statistical analyses in the RI context, *Emx1-Nrxn2* cKO mice did not exhibit significant changes in response rate during the CRF1 sessions [RM two-way ANOVA: F (1,14) = 1.258, *p* = 0.2808] (Fig. 2b). During the scheduled reinforcement in the RI context, *Emx1-Nrxn2* cKO mice did not exhibit any significant change in total number of lever presses [RM two-way ANOVA: F (1,14) = 0.6249, *p* = 0.4424] (Fig. 2c) or response rate [RM two-way ANOVA: F (1,14) = 0.7990, *p* = 0.3865] (Fig. 2d) or reward rate [RM two-way ANOVA: F (1,14) = 0.9837, *p* = 0.3381] (Fig. 2e). *Emx1-Nrxn*2 cKO mice did not exhibit significant changes in response rate during the CRF1 sessions in the RR context [RM two-way ANOVA: F (1,14) = 0.6432, *p* = 0.4320] (Fig. 2f). During the scheduled reinforcement in the RR context, *Emx1-Nrxn2* cKO mice exhibited alterations in total number of lever presses [RM two-way ANOVA: F (1,14) = 5.128, *p* < 0.05] (Fig. 2g) that was day-dependent [RM two-way ANOVA: F (5,70) = 2.770, *p* < 0.05] but not in response rate [RM two-way ANOVA: F (1,14) = 1.133, *p* = 0.3051] (Fig. 2h) or reward rate [RM two-way ANOVA: F (1,14) = 2.060, *p* = 0.1732] (Fig. 2i).. In summary, these results suggest that *Emx1Cre* driven deletion of *Nrxn2* did not affect instrumental responding behavior in a context that promotes either goal-directedness or habitual behavior.

### *Emx1Cre* driven deletion of *Nrxn2* impaired the ability of mice to discriminate between goal-directed and habitual action strategies in response to outcome devaluation

**Figure 3:**
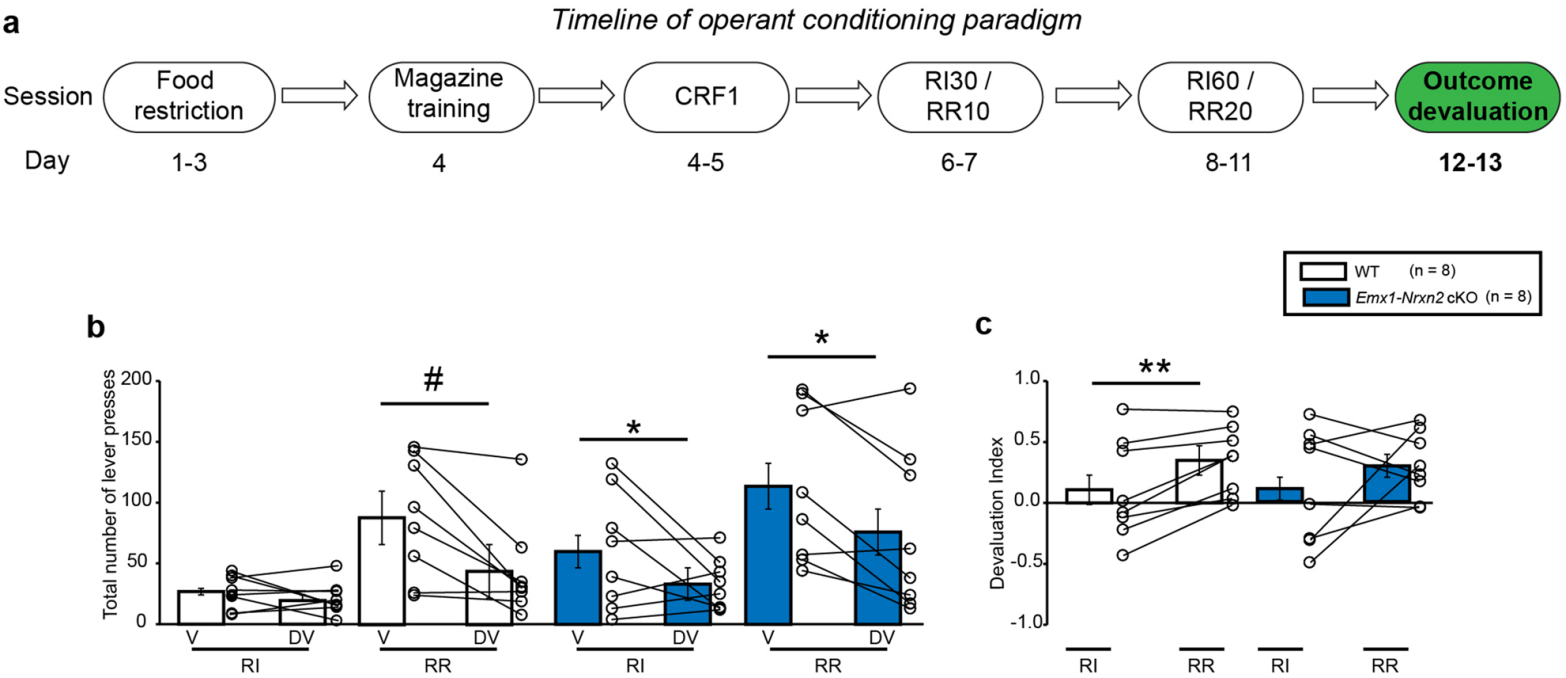
*Emx1-Nrxn2* cKO mice failed to process contextual information to bias action control in response to outcome devaluation. (**a**) Experimental timeline of outcome devaluation in mice. Mice underwent the outcome devaluation procedure on days 12–13. On valued day, mice had 1 h ad libitum access to 20% sucrose in their home cage, followed by a brief, non-reinforced test sessions for 5 min in the random interval (RI) and random ratio (RR) contexts. On the devalued day, mice had 1 h ad libitum access to 20 mg food pellets that were previously earned in the operant chambers followed by brief, non-reinforced 5 min test sessions in both the RI and RR contexts. (**b**–**c**) WT mice processed contextual information to bias action control in response to outcome devaluation. WT mice were sensitive to outcome devaluation in a context-dependent manner [RM two-way ANOVA: F (1,14) = 7.240, *p* < 0.05]. Sidak’s post hoc test confirmed that WT mice reduced their lever presses on the devalued (DV) versus valued (V) day in the RR (t = 4.338; *p* < 0.01), but not in the RI (t = 0.5330; *p *= 0.8419) context (**b**) that was accompanied by a higher devaluation index in the RR context versus the RI context (df = 7, *p* < 0.01, two-tailed Student’s *t*-test) (**c**). *Emx1-Nrxn2* cKO mice failed to process contextual cues to bias action control in response to outcome devaluation. *Emx1-Nrxn2* cKO mice exhibited similar lever pressing behavior between V and DV days in RI and RR contexts [RM two-way ANOVA: F (1,14) = 0.2994, *p* = 0.5929] (**b**) without any shift in devaluation index between both the contexts (df = 7, *p *= 0.3681, two-tailed Student’s *t*-test) (**c**). Data is represented as means ± SEM for 8 mice per genotype. Each open circle in the summary graphs represents each mouse. Statistical assessments were performed by RM two-way ANOVA (**b**) followed by Sidak’s post hoc test by comparing V to DV in each context with *p < 0.05 or #*p* < 0.05. Student’s *t*-test (**c**) was used to compare RI to RR context with *p < 0.05. Symbols of statistical significance derived from RM two-way ANOVA or Student’s *t*-test analyses were denoted by (*) and symbols of statistical significance derived from Sidak’s post hoc test were denoted by (#). The green color in the experimental timeline (**a**) corresponds to the operant conditioning data being represented in (**b**–**c**).

During the outcome devaluation phase on day 12–13 (Fig. 3a), RM ANOVA detected a significant effect of devaluation state [RM two-way ANOVA: F (1,14) = 11.86, *p* < 0.01] and context [RM two-way ANOVA: F (1,14) = 7.594, *p* < 0.05] on lever pressing behavior in WT mice. There was also a significant devaluation state × context interaction [RM two-way ANOVA: F (1,14) = 7.240, *p* < 0.05] indicating that sensitivity of WT mice to outcome devaluation was context-dependent. Sidak’s post hoc test confirmed that WT mice significantly reduced their lever pressing behavior on the devalued day v/s valued day in the RR context (t = 4.338; *p* < 0.01) but not in the RI context (t = 0.5330; *p* = 0.8419) (Fig. 3b). Additionally, WT mice were able to shift between two action selection strategies as indicated by a significant change in devaluation index between RI and RR contexts (df = 7, *p* < 0.01, two-tailed Student’s *t*-test) (Fig. 3c). In the *Emx1-Nrxn2* cKO mice, there was a significant effect of devaluation state [RM two-way ANOVA: F (1,14) = 10.41, *p* < 0.01] but a non-significant trend towards effect of context [RM two-way ANOVA: F (1,14) = 3.812, *p* = 0.0712] on lever pressing behavior. There was no significant devaluation state × context interaction [RM two-way ANOVA: F (1,14) = 0.2994, *p* = 0.5929] indicating that the *Emx1-Nrxn2* cKO mice unable to distinguish between goal-directed and habitual action strategies in response to outcome devaluation (Fig. 3b). In contrast to WT mice, *Emx1-Nrxn2* cKO mice were unable to shift between goal-directed and habitual action strategies as indicated by lack of change in devaluation index between RI and RR contexts (df = 7, *p* = 0.3681, two-tailed Student’s *t*-test) (Fig. 3c). Overall, the data suggests that the *Emx1-Nrxn2* cKO mice failed to integrate contextual cues with the appropriate action selection strategy to make informed decisions in response to outcome devaluation.

### *Nrxn2* deletion in Cg1/M2 cortex increased instrumental responding in the habitual context in mice

**Figure 4:**
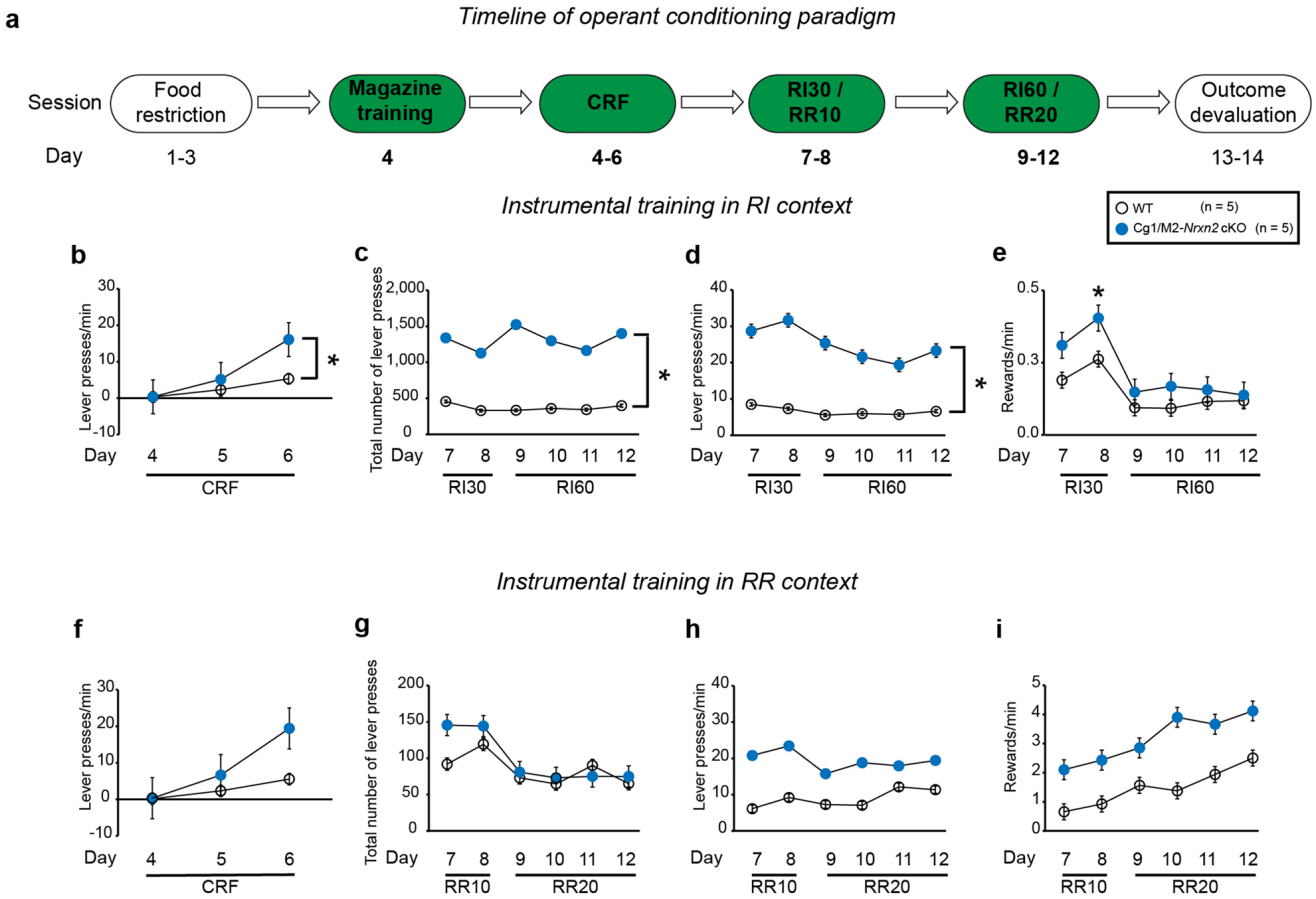
Cg1/M2 *Nrxn2* cKO mice exhibited increased instrumental responding behavior in a context that promotes habitual behavior. (**a**) Experimental timeline of instrumental training in mice. Mice underwent random time (RT) schedule on day 4. This was followed by continuous ratio of reinforcement (CRF) sessions on days 4–6. Mice underwent CRF1 (1 lever press = 1 reward) on day 4, CRF5 (5 lever presses = 1 reward) on day 5 and CRF15 (15 lever presses = 1 reward) on day 6. Following CRF sessions, mice underwent random interval (RI) and random ratio (RR) schedules of reinforcement on days 7–12 in a context-specific manner. On days 7–8, mice pressed the lever under RI30, wherein passing of 30 s had a 15% probability of pellet dispensation, and RR10 wherein every lever press had a 10% probability of pellet dispensation. This was followed by RI60 and RR20 sessions on days 9–12. Following the RI and RR training sessions, mice were provided with ad libitum access to a 20% sucrose solution in their home cage. (**b**–**e**) Cg1/M2 *Nrxn2* cKO mice exhibited altered instrumental responding behavior in the RI context. Cg1/M2 *Nrxn2* cKO mice exhibited increases in response rate (lever presses/min) [F (1,8) = 5.443, *p* < 0.05] during the CRF sessions (**b**), total number of lever presses [F (1,8) = 6.030, *p* < 0.05] (**c**), response rate (lever presses/min) [F (1,8) = 6.165, *p* < 0.05] (**d**), and reward rate (rewards/min) [F (1,18) = 6.785, *p* < 0.05] (**e**) during the 6 RI sessions.  (**f**–**i**) Cg1/M2 *Nrxn2* cKO exhibited normal instrumental responding behavior in the RR context. Cg1/M2 *Nrxn2* cKO mice exhibited a trend towards increased response rate (lever presses/min) [F (1,8) = 5.184, *p* = 0.0523] (**f**) during the 3 CRF sessions in the random ratio (RR) context. Cg1/M2 *Nrxn2* cKO mice did not exhibit any significant changes in total number of lever presses [F (1,8) = 2.645, *p* = 0.1425] (**g**), but there was a non-significant trend towards increased response rate (lever presses/min) [F (1,8) = 4.153, *p* = 0.0759] (**h**) and reward rate (rewards/min) [F (1,8) = 4.505, *p* = 0.0666] (**i**) during the 6 RR sessions. Each circle in the time-course plots (**b**–**i**) represents means ± SEM for 5 mice per genotype. Statistical assessments were performed by RM two-way ANOVA (**b**–**i**) by comparing WT to Cg1/M2 *Nrxn2* cKO mice with *p < 0.05. The green color in the experimental timeline (**a**) corresponds to the operant conditioning data being represented in (**b**–**i**).

To gain specific understanding of role of Nrxn2 in the balance between goal-directed and habitual actions, we conditionally deleted *Nrxn2* in the Cg1/M2 cortex in male adult *Nrxn2*^*f/f*^ mice that has been implicated in goal-directed actions. WT and *Cg1/M2 Nrxn2* cKO mice underwent RI (to promote habitual behavior) and RR (to promote goal-directed behavior) in two separate contexts (Fig. 4a). Upon doing within-group statistical comparisons in WT mice, there was no significant effect of context [RM two-way ANOVA: F (1,8) = 0.0009, *p* = 0.9923] on response rate during the CRF sessions. There was no significant effect of context on total number of lever presses [RM two-way ANOVA: F (1,8) = 3.051, *p* = 0.1188] and on response rate [RM two-way ANOVA: F (1,8) = 0.2291, *p* = 0.6450] but the effect was trending towards significance for reward rate [RM two-way ANOVA: F (1,8) = 4.729, *p* = 0.0614] during the scheduled reinforcement phase in WT mice. Upon doing within-group statistical analyses in Cg1/M2 *Nrxn2* cKO mice, there was no significant effect of context on response rate [RM two-way ANOVA: F (1,8) = 0.3124, *p* = 0.5915] during the CRF sessions. There was a significant effect of context on total number of lever presses [RM two-way ANOVA: F (1,8) = 12.25, *p* < 0.01], on reward rate [RM two-way ANOVA: F (1,8) = 35.75, *p* < 0.001] but not on response rate [RM two-way ANOVA: F (1,8) = 0.5362, *p* = 0.4849] during the scheduled reinforcement phase in the Cg1/M2 *Nrxn2* cKO mice.

Upon doing between-group statistical analyses, Cg1/M2 *Nrxn2* cKO mice exhibited a significantly higher response rate during the CRF sessions in the RI context [RM two-way ANOVA: F (1,8) = 5.443, *p* < 0.05] (Fig. 4b) with significant day × genotype interaction [RM two-way ANOVA: F (2,16) = 7.124, *p* < 0.01]. During the scheduled reinforcement phase in the RI context, Cg1/M2 *Nrxn2* cKO mice exhibited a significantly higher total number of lever presses [RM two-way ANOVA: F (1,8) = 6.030, *p* < 0.05] (Fig. 4c) along with an increase in response rate [RM two-way ANOVA: F (1,8) = 6.165, *p* < 0.05] (Fig. 4d) and reward rate [RM two-way ANOVA: F (1,18) = 6.785, *p* < 0.05] (Fig. 4e) in comparison to WT mice. There was no significant day × genotype interaction for total number of lever presses [RM two-way ANOVA: F (5,40) = 1.215, *p* = 0.3197] and response rate [RM two-way ANOVA: F (5,40) = 1.705, *p* = 0.1557] but this interaction effect was trending towards statistical significance for reward rate [RM two-way ANOVA: F (5,40) = 2.162, *p* = 0.0777] in the RI context. During the conditioning phase in the RR context, Cg1/M2 *Nrxn2* cKO mice tended to exhibit higher response rate than WT mice [RM two-way ANOVA: F (1,8) = 5.184, *p* = 0.0523] during the CRF sessions (Fig. 4f) and their response rate was day-dependent as indicated by a significant day × genotype interaction [RM two-way ANOVA: F (2,16) = 5.716, *p* < 0.05]. During the scheduled reinforcement in RR context, Cg1/M2 *Nrxn2* cKO mice did not show any changes in total number of lever presses [RM two-way ANOVA: F (1,8) = 2.645, *p* = 0.1425] (Fig. 4g) but they exhibited a non-significant trend towards higher response rate [RM two-way ANOVA: F (1,8) = 4.153, *p* = 0.0759] (Fig. 4h) and reward rate [RM two-way ANOVA: F (1,8) = 4.505, *p* = 0.0666] (Fig. 4i). There was no significant day × genotype interaction for total number of lever presses [RM two-way ANOVA: F (5,40) = 1.660, *p* = 0.1666], response rate [RM two-way ANOVA: F (5,40) = 1.891, *p* = 0.1175] and reward rate [RM two-way ANOVA: F (5,40) = 0.6158, *p* = 0.6884] during the scheduled reinforcement in the RR context. Overall, the results suggest that *Nrxn2* deletion in the Cg1/M2 cortex enhanced instrumental responding behavior in a context-dependent manner.

### *Nrxn2* deletion in Cg1/M2 cortex impaired lever press distribution in response to outcome devaluation in the goal-directed context in mice

**Figure 5:**
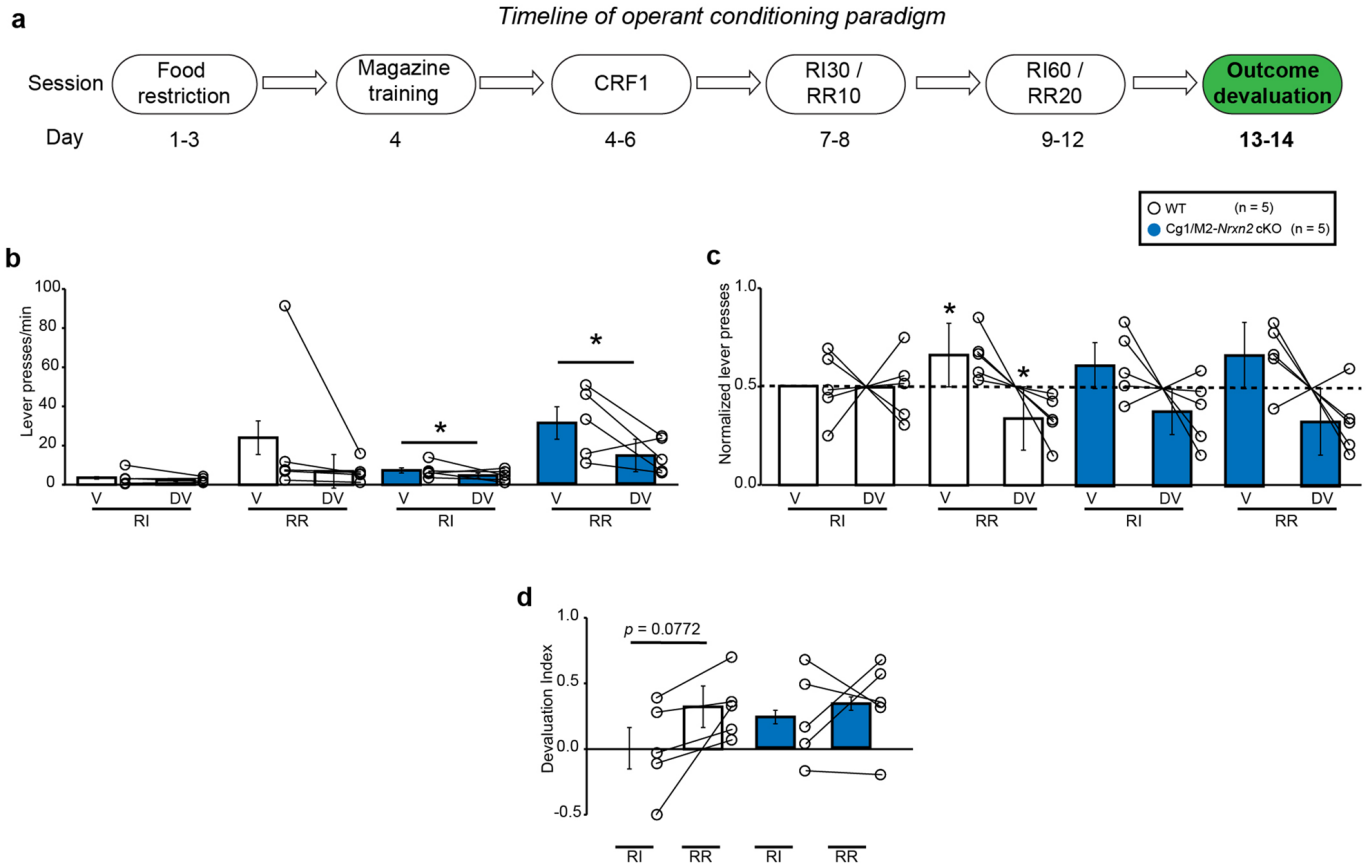
Cg1/M2 *Nrxn2* cKO mice failed to differentially distribute their lever presses in response to outcome devaluation in a context that promotes goal-directedness. (**a**) Experimental timeline of outcome devaluation in mice. Mice underwent the outcome devaluation procedure on days 12–13. On valued day, mice had 1 h ad libitum access to 20% sucrose in their home cage, followed by a brief, non-reinforced test sessions for 5 min in the random interval (RI) and random ratio (RR) contexts. On the devalued day, mice had 1 h ad libitum access to 20 mg food pellets that were previously earned in the operant chambers followed by brief, non-reinforced 5 min test sessions in both the RI and RR contexts. (**b**–**d**) WT mice exhibited goal-directedness in response to outcome devaluation. WT mice did not reduce their lever pressing behavior in response to outcome devaluation in both RI and RR contexts [RM two-way ANOVA: F (1,8) = 1.187, *p* = 0.3077] (**b**). WT mice significantly distributed their lever presses between valued (V) and devalued days (DV) in the RR context (valued state = 0.66, devalued state = 0.34; *p* < 0.05, one sample t-test) but not in the RI context (valued state = 0.5, devalued state = 0.5; *p* > 0.9999, one sample t-test) (**c**). This was accompanied by a non-significantly higher devaluation index in the RR context versus RI context (df = 4, *p* = 0.0772, two-tailed Student’s *t*-test) (**d**).  Cg1/M2 *Nrxn2* cKO mice failed to distribute their lever presses between V and DV days in response to outcome devaluation in the RR (valued state = 0.67, devalued state = 0.33; *p* = 0.0900, one sample t-test) and RI contexts (valued state = 0.62, devalued state = 0.38; *p* = 0.2094, one sample t-test) (**c**) without any shift in devaluation index between both the contexts (df = 4, *p* = 0.5990, two-tailed Student’s *t*-test) (**d**). Data is represented as means ± SEM for 5 mice per genotype. Each open circle in the summary graphs represents each mouse. Statistical assessments were performed by RM two-way ANOVA by comparing V to DV in each context within each genotype with *p < 0.05 (**b**) or one-sample t-test by comparing normalized lever presses within each context against a “no devaluation” point of 0.5 with *p < 0.05 (**c**) or Student’s *t*-test (**d**). The green color in the experimental timeline (**a**) corresponds to the operant conditioning data being represented in (**b**–**d**).

During the outcome devaluation phase on day 12–13 (Fig. 5a), there was no significant effect of either devaluation state [RM two-way ANOVA: F (1,8) = 1.556, *p* = 0.2475] or context [RM two-way ANOVA: F (1,8) = 1.683, *p* = 0.2307] on lever pressing behavior in WT mice. There was no significant devaluation state × context interaction [RM two-way ANOVA: F (1,8) = 1.187, *p* = 0.3077] on lever pressing behavior in WT mice (Fig. 5b). The change in devaluation index between RI and RR context trended towards statistical significance (df = 4, *p* = 0.0772, two-tailed Student’s *t*-test) in WT mice indicating that these mice tended to shift between goal-directed and habitual action strategies (Fig. 5d). In the Cg1/M2 *Nrxn2* cKO mice, there was a significant effect of devaluation state [RM two-way ANOVA: F (1,8) = 5.722, *p* < 0.05] and context [RM two-way ANOVA: F (1,8) = 10.77, *p* < 0.05] on lever pressing behavior. There was no significant devaluation state × context interaction [RM two-way ANOVA: F (1,8) = 3.021, *p* = 0.1204] in Cg1/M2 *Nrxn2* cKO mice (Fig. 5b). Cg1/M2 *Nrxn2* cKO mice did not exhibit any shift between goal-directed and habitual action strategies as indicated by lack of significant change in devaluation index between RR and RI contexts (df = 4, *p* = 0.5990, two-tailed Student’s *t*-test) (Fig. 5d).

Due to lack of significant changes in lever pressing rate in control mice in response to outcome devaluation the RR context, we analyzed the distribution of lever presses between valued and devalued states in both RR and RI contexts in WT and Cg1/M2 *Nrxn2* cKO mice. A one-sample t-test (against a “no devaluation” point or 0.5) of normalized lever presses between valued and devalued states in the RI context showed that both WT (valued state = 0.5, devalued state = 0.5; *p* > 0.9999, one sample t-test) (Fig. 5c) and Cg1/M2 *Nrxn2* cKO mice (valued state = 0.62, devalued state = 0.38; *p* = 0.2094, one sample t-test) (Fig. 5c) exhibited similar distribution of lever presses between valued and devalued states indicating that both WT and Cg1/M2 *Nrxn2* cKO mice adopted a habitual action strategy. In the RR context, the WT mice (valued state = 0.66, devalued state = 0.34; *p* < 0.05, one sample t-test) (Fig. 5c) adopted a goal-directed strategy as indicated by a higher preference for lever pressing in valued v/s devalued state. Cg1/M2 *Nrxn2* cKO mice (valued state = 0.67, devalued state = 0.33; *p* = 0.0900, one sample t-test) failed to adopt a goal-directed strategy, although this trend was trending towards statistical significance (Fig. 5c). Thus, *Nrxn2* deletion in the Cg1/M2 cortex tended to impair goal-directed action strategy.

### *Emx1-Cre* driven deletion of *Nrxn2* induced reversal learning deficits in mice without any impairments in spatial navigation

**Figure 6:**
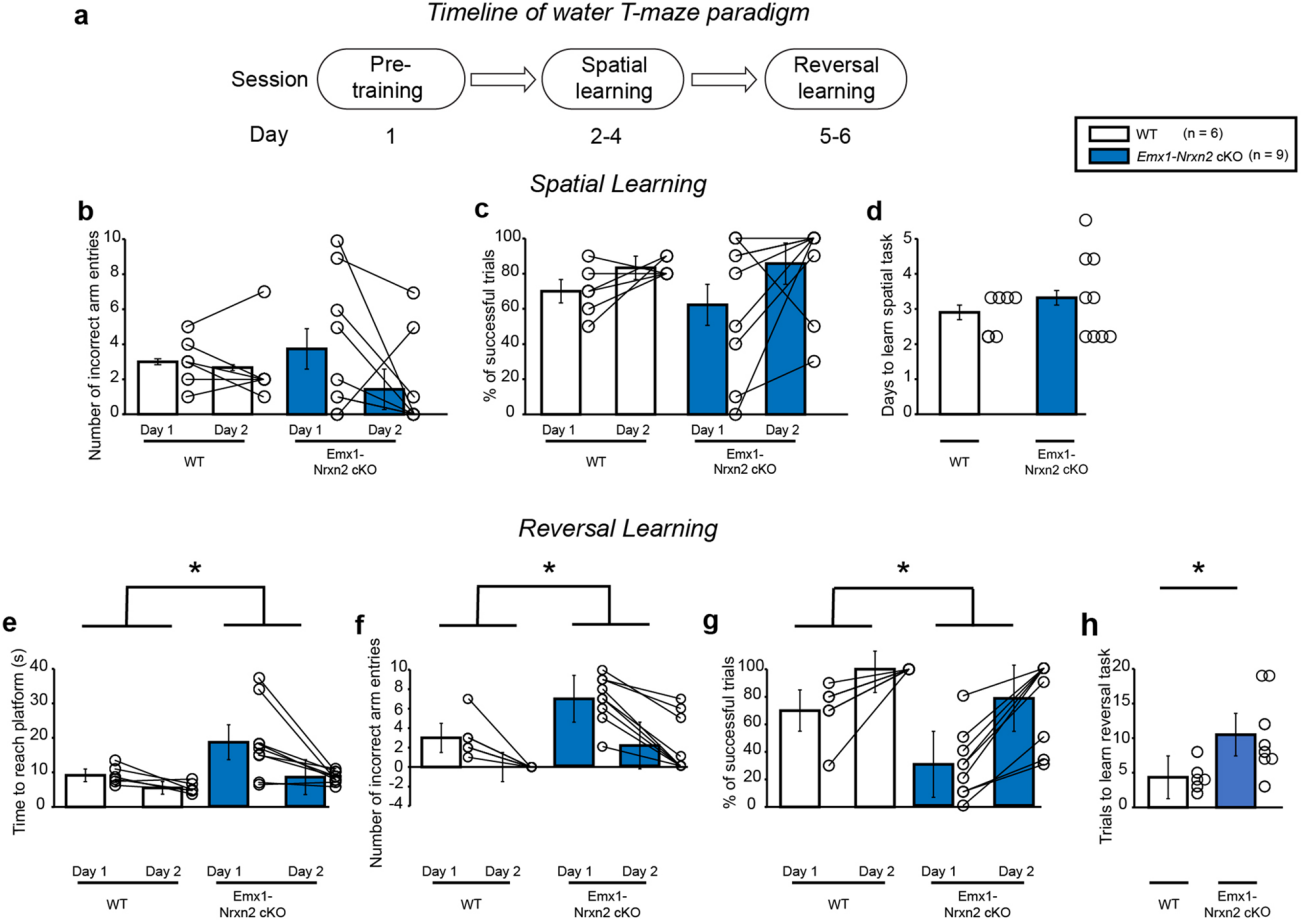
*Emx1-Nrxn2* cKO mice exhibited reversal learning deficits in a water T-maze paradigm. (**a**) Experimental timeline of water T-maze paradigm in mice. Mice underwent a pre-training session on day 1. This was followed by a training session in which mice were subjected to 10 trials / day. Upon completion of 8 successful trials for 2 consecutive days, mice proceeded to the reversal learning session. For reversal learning session, the platform was placed in the arm opposite to the one chosen during training session. Mice were subjected to 10 trials / day for a total period of 2 days. (**b**–**d**) *Emx1-Nrxn2* cKO mice exhibited normal spatial navigation. *Emx1-Nrxn2* cKO mice did not exhibit any significant changes in number of incorrect arm entries, [RM two-way ANOVA: F (1,13) = 0.06481, *p* = 0.8030] (**b**) or % of successful trials during the spatial task on days 1 and 2 [RM two-way ANOVA: F (1,13) = 0.06481, *p* = 0.8030] (**c**). *Emx1-Nrxn2* cKO mice took similar number of days taken to learn the spatial task in comparison to control mice (df = 13, *p *= 0.5100, two-tailed Student’s *t*-test) (**d**). (**e**–**h**) *Emx1-Nrxn2* cKO mice exhibited reversal learning deficits. *Emx1-Nrxn2* cKO mice took significantly longer time to reach platform during reversal learning task on days 1 and 2 [RM two-way ANOVA: F (1,13) = 6.562, *p* < 0.05] (**e**). *Emx1-Nrxn2* cKO mice made significantly more incorrect arm entries. [F (1,13) = 7.841, *p* < 0.05] (**f**) and exhibited significantly lower % of successful trials during the reversal learning task on days 1 and 2 [F (1,13) = 7.841, *p* < 0.05] (**g**). *Emx1-Nrxn2* cKO took significantly more trials to learn the reversal task (df = 9.195, *p* < 0.05, two-tailed Student’s *t*-test with Welch’s correction) (**h**). Data is represented as means ± SEM for 6 WT and 9 *Emx1-Nrxn2* cKO mice. Each open circle in the summary graphs represents each mouse. Statistical assessments were performed by RM two-way ANOVA (**b**, **c**, **e**–**g**) or two-tailed Student’s *t*-test (**d**, **h**) by comparing WT to *Emx1-Nrxn2* cKO mice with *p < 0.05.

During the spatial navigation phase (Fig. 6a), *Emx1-Nrxn2* cKO mice did not exhibit any significant changes in number of incorrect arm entries [RM two-way ANOVA: F (1,13) = 0.06481, *p* = 0.8030] (Fig. 6b) and had a similar % of successful trials to WT mice [RM two-way ANOVA: F (1,13) = 0.06481, *p* = 0.8030] (Fig. 6c). There was no significant day × genotype interaction for either number of incorrect arm entries [RM two-way ANOVA: F (1,13) = 0.3093; *p* = 0.5876] or % of successful trials [RM two-way ANOVA: F (1,13) = 0.3093; *p* = 0.5876]. There was also no significant difference in number of days to learn spatial task between WT and *Emx1-Nrxn2* cKO mice (df = 13, *p* = 0.5100, two-tailed Student’s *t*-test) (Fig. 6d). Hence, *Emx1Cre* driven deletion of *Nrxn2* did not disrupt spatial learning in mice.

During the reversal learning phase (Fig. 6a), *Emx1-Nrxn2* cKO mice took significantly more time to reach the platform [RM two-way ANOVA: F (1,13) = 6.562, *p* < 0.05] (Fig. 6e). *Emx1-Nrxn2 cKO* mice made more incorrect arm entries [RM two-way ANOVA: F (1,13) = 7.841, *p* < 0.05] (Fig. 6f) and had a lower % of successful trials than WT mice [RM two-way ANOVA F (1,13) = 7.841, *p* < 0.05] (Fig. 6g) indicating reversal learning deficits in *Emx1-Nrxn2* cKO mice. There was no significant day × genotype interaction for either time to reach platform [F (1,13) = 2.264, *p* = 0.1563] or number of incorrect arm entries [RM two-way ANOVA: F (1,13) = 2.835, *p* 0.1160) or % of successful trials [RM two-way ANOVA: F (1,13) = 2.835, *p* = 0.1160] indicating that *Emx1-Nrxn2* cKO exhibited behavioral deficits across both days of reversal learning. Lastly, *Emx1-Nrxn2* cKO mice took significantly more trials than WT mice to consistently learn the reversal learning task (df = 9.195, *p* < 0.05, two-tailed Student’s *t*-test with Welch’s correction) (Fig. 6h). Overall, the *Emx1-Nrxn2* cKO mice exhibited reversal learning deficits in a day-independent manner.

## Discussion

Our studies provide the first evidence that deletion of *Nrxn2* under two distinct conditional approaches can disrupt complex learning strategies that can underlie flexible behavior in a dynamic environment. We demonstrated that *Nrxn2* deletion impaired the ability to alternate between goal-directed and habitual action strategies in response to alterations in A–O contingencies.

The instrumental learning behavior of *Emx1-Nrxn2* cKO mice was intact indicating that *Nrxn2* deletion did not perturb habit formation. *Emx1-Nrxn2* cKO mice failed to discriminate between contexts upon devaluation of A–O contingencies in which they had to either adopt a goal-directed or habitual action strategy. Although *Emx1-Nrxn2* cKO mice did not exhibit deficits in S–R learning in the RI context, they were still sensitive to outcome devaluation in the same context, suggesting habitual responding impairments in these mice. Consequentially, the *Emx1-Nrxn2* cKO mice were unable to shift between goal-directed and habitual action strategies as indicated by lack of change in devaluation index between RI and RR contexts.

The failure to process contextual information to make informed decisions was also replicated in the water T-maze test. *Emx1-Nrxn2* cKO mice had normal spatial learning function but had reversal learning deficits. This data suggests that the *Emx1-Nrxn2* cKO mice were unable to integrate novel contextual cues with consequences of action that would necessitate them from disengaging from preferred behavioral patterns and learning new ones. These mice also took a longer time in finding the new location of the platform during the reversal learning phase indicating they were slower than the control mice in processing novel contextual information to make goal-directed decisions. The *Emx1-Nrxn2* cKO mice took significantly more trials than the control mice to consistently learn the reversal of A–O contingencies in the water T-maze task. This behavior could be termed as “perseverative”^42,43^ since *Emx1-Nrxn2* cKO mice continued eliciting the same body action in the reversal learning phase that they previously learned during the spatial training phase. The argument that *Emx1-Nrxn2* cKO mice exhibit perseverative behavior is in line with previous findings where *Nrxn2* cKO mice were reported to exhibit increased nestlet shredding^21^ and grooming behaviors^22^. It is plausible that these mice tend to over-rely on actions that are established through S–R learning, and continue performing those actions in situations where they are deemed unnecessary, leading to manifestation of a ritualistic-type behavior. However, this argument of perseverative behavior does not explain the reduced lever pressing behavior displayed by the *Emx1-Nrxn2* cKO mice in the RI and RR contexts in response to outcome devaluation. Hence, it appears that *Emx1-Nrxn2* cKO mice are unable to disengage from habits that were previously learned through hippocampal-dependent spatial-cognitive tasks but can still deviate from habits that are acquired through striatal-dependent response learning.

An interesting observation from the water T-maze test is that the *Emx1-Nrxn2* were able to consistently perform the new action throughout the remainder of the reversal learning phase after they learned the contingency reversal. In other words, these mice did not exhibit any “regressive behavior” suggesting that they do not encounter any difficulties in maintaining the new behavioral pattern^42,43^. This lack of regressive behavior could potentially be explained by previous findings where *Emx1-Nrxn2* cKO mice exhibited more immature synapses than those of WT mice without any changes in mature spines or dendritic branching in hippocampal CA1 region^21^. There was also a non-significant trend towards higher ratio of immature spines to mature spines in *Emx1-Nrxn2* cKO mice^21^. Interestingly, conditional deletion of *Nrxn2* through Cre recombinase injection in the CA1 region of *Nrxn2*^*f/f*^ mice enhanced CA3-CA1 synaptic connections^23^. *Nrxn2* deletion has been shown to increase excitatory synaptic density^23^. The increased synaptic density (mature or immature) could actively engage in repetitive learning to form stable synaptic connections. Together with our spine morphology data suggests that the reversal learning deficits in *Emx1-Nrxn2* cKO mice are attributed to the inability to disengage from preferred behavioral patterns and not due to the inability to learn and maintain those new behavioral patterns.

In contrast to *Emx1-Nrxn2* cKO mouse model, *Nrxn2* deletion under the control of Cre recombinase in the Cg1/M2 cortex increased instrumental responding behavior in the RI context indicating a higher tendency for habit formation in the Cg1/M2 *Nrxn2* cKO mice. Despite the higher S–R learning behavior in the RI context displayed by Cg1/M2 *Nrxn2* cKO mice, they were still able to disengage from their habitual behavior in response to outcome devaluation in the RI context as indicated by the significant effect of devaluation state on lever pressing behavior. When we analyzed the distribution of lever presses between valued and devalued days, we failed to observe any distinction in lever pressing behavior between both in response to outcome devaluation in the RR context suggesting impairments in goal-directedness in these mice. *Nrxn2* deletion from the Cg1/M2 circuitry could affect the synaptic connectivity and functionality of the Cg1/M2 innervated DMS circuitry. This would compel the mice to rely on the habitual circuitry to learn the instrumental task. This could potentially explain the higher instrumental learning in the Cg1/M2 *Nrxn2* cKO mice in the RI context that promotes habitual responding and the lack of differential distribution of lever presses upon outcome devaluation in the RR context.

The molecular mechanisms by which *Nrxn2* deletion can disrupt reversal learning or integration of contextual knowledge to update action control remains to be understood. Modulation of dopaminergic activity by pharmacological approaches have been reported to be involved in reversal learning^44–46^. Stimulation of dopamine D2, but not D1 receptors, in the nucleus accumbens core disrupted reversal learning^45^. In a recent study, pan deletion of all three *Nrxn* genes (Nrxn1/2/3) in mice (referred to as “pan-Nrxn cKO mice”) from dopamine (DA) neurons increased densities of dopamine transporter (DAT) and vesicular monoamine transporter (VMAT2) in the ventral striatum, increased GABA release from DA terminals, and reduced DA overflow upon blockade of nicotinic receptors indicating that Nrxns may be required for DA and GABA signaling in DA neurons^8^. At the behavioral level, amphetamine-induced locomotor activity was attenuated in pan-Nrxn cKO mice without altering other dopaminergic-based behaviors^8^. To date, the effects of *Nrxn2* deletion on DA neurotransmission and associated behaviors is not known. Hence, similar approaches that conditionally deletes *Nrxn2* from DA neurons in the dorsal striatum would be warranted to provide novel mechanistic insights by which *Nrxn2* deletion can induce decision-making deficits.

Individuals with *NRXN2* mutations exhibited autistic features including speech and language impairments^10,11^. Whether these individuals can learn the contingencies between action and outcome to make meaningful decisions in a dynamic environment is not known. The findings from our preclinical studies suggest that individuals with *NRXN2* mutations can utilize spatial-cognitive information or S–R information to form actions but may have more challenges in updating their actions to make more meaningful goal-directed decisions under circumstances where those habits need to be changed or updated. Overall, our studies will begin to establish a role for Nrxn2 in the manifestation of cognitive rigidity that is associated with several neuropsychiatric disorders in which *NRXN2* mutations have been documented including ASD.

## Data Availability

The raw data for this study is available in the supplementary section on the journal’s website. Further inquiries can be directed to the corresponding author.
